# Intraoperative lumbar drainage can prevent cerebrospinal fluid leakage during transsphenoidal surgery for pituitary adenomas: a systematic review and meta-analysis

**DOI:** 10.1186/s12883-020-01877-z

**Published:** 2020-08-15

**Authors:** Jiahe Tan, Rui Song, Renzheng Huan, Ning Huang, Jin Chen

**Affiliations:** 1 Department of Neurosurgery, The Second Affiliated Hospital of Chongqing Medical University, Chongqing, 400010 China; 2 Department of Gastroenterology, The Second Affiliated Hospital of Chongqing Medical University, Chongqing, 400010 China

**Keywords:** Lumbar drainage, Cerebrospinal fluid leakage, Pituitary adenoma, Transsphenoidal surgery, Meta-analysis

## Abstract

**Background:**

Perioperative cerebrospinal fluid (CSF) leakage is a major complication of pituitary adenomas transsphenoidal surgery. Lumbar drainage (LD) is a common method of treating CSF leakage. But whether intraoperative LD can prevent CSF leakage during the perioperative period of pituitary adenomas transsphenoidal surgery remains controversial. Clarity on the appropriate use of LD is needed.

**Methods:**

A systematic literature review was conducted in the PubMed, EMBASE, and Web of science databases. Articles were included when they compared intraoperative LD with intraoperative no-LD CSF leakage rates during pituitary adenomas transsphenoidal surgery.

**Results:**

Overall, 5 studies containing 678 cases met the inclusion criteria. When data were provided on intraoperative CSF leakage rates, the meta-analysis showed a significant difference in favor of intraoperative LD. When data were provided on postoperative CSF leakage rates, the meta-analysis also demonstrated a significant difference in favor of intraoperative LD.

**Conclusions:**

Although the results of this meta-analysis suggest intraoperative LD can reduce the risk of CSF leakage during the perioperative period of pituitary adenomas transsphenoidal surgery, the available evidence is indefinite. To some extent the results suggest intraoperative LD’s potential positive role. Further studies that include well-designed prospective, randomized controlled clinical trials are necessary for further verification.

## Background

Pituitary adenoma is a common intracranial tumor. The prevalence of pituitary adenoma ranges from 1 in 865 to 1 in 2688 according to a review published in JAMA [1]. When the patient has clear indications for surgery, transsphenoidal surgical adenoma resection is the preferred treatment. Both endoscopic and microsurgical transsphenoidal surgery are commonly performed, while craniotomy is rarely performed [2, 3]. Perioperative cerebrospinal fluid (CSF) leakage is one of the major complications associated with transsphenoidal surgery [4]. The occurrence of intraoperative CSF leakage is associated with many factors, such as surgical technique, the tumor aggressiveness, tumor volume and location, and the tumor’s relationship with the surrounding neurovascular structures. Primary reconstruction of the skull base is the most important surgical technique [5]. Postoperative CSF leakage occurs due to failure to recognize an intraoperative CSF leakage or a failure of the primary repair [6], which is conservatively managed with lumbar drainage (LD) for 3–5 days or with surgical repair [7].

Recently, one review and one meta-analysis showed that preoperative or intraoperative LD played no apparent role in preventing postoperative CSF leakage during endoscopic skull base lesions surgery [8, 9]. Another review showed that recent studies had not shown encouraging results with the use of LD in preventing CSF leakage during the perioperative period of pituitary adenomas transsphenoidal surgery [10]. While recent studies reported that intraoperative LD is related to CSF leakage rate during the perioperative period of pituitary adenomas transsphenoidal surgery [11–15], but the relevant results are different. The conflicting results complicated attempts to elucidate whether or not intraoperative LD prevented CSF leakage. We realized that there was no meta-analysis to compare intraoperative LD with no intraoperative LD for preventing CSF leakage during the perioperative period of pituitary adenomas transsphenoidal surgery. To clarify this, we conducted our meta-analysis.

## Methods

A systematic review of the literature and a meta-analysis were conducted by following the Preferred Reporting Items for Systematic Reviews and Meta-Analysis (PRISMA) guidelines [16].

### Literature search

We conducted a comprehensive literature search in the PubMed, EMBASE, and Web of Science databases to evaluate the association between intraoperative LD and CSF leakage during the perioperative period of pituitary adenomas transsphenoidal surgery. Search terms included “lumbar drainage,” “cerebrospinal fluid leakage,” and “pituitary adenoma,” in Medical Subject Headings (MeSh) terms with their entry terms’ appropriate synonyms. The literature search period ended on April 25, 2020.

### Inclusion and exclusion criteria

We included the articles using the following criteria according to evidence-based medicine literature retrieval format: (1) Population: patients were pathologically diagnosed with pituitary adenoma according to postoperative immunocytology. And the operative approach was via the sphenoidal sinus using endoscope or microscope. (2) Interventions: LD was placed before the surgery and then used for CSF drainage during the perioperative period. (3) Comparisons: LD was not used intraoperatively. (4) Outcomes: studies had data and endpoints on the CSF leakage. As for the definition of CSF leakage, it was a discontinuous or continuous flow of clear fluid from the sellar diaphragm (intraoperation) or nasal cavity (postoperation). Meanwhile, clinical symptoms, experience of doctors and detection of β2-transferrin in fluid outflow were evaluated for CSF leakage. (5) Other criteria: operators used the appropriate methods in that period to reconstruct the skull base. And time span of perioperative period ranged from the day of surgery to 1 week postoperatively. The exclusion criteria were as follows: (1) Repetitive articles were excluded. (2) The priority for selection was the randomized controlled trials (RCTs) and the cohort studies, other literature studies were excluded. (3) LD was placed after a clear CSF leakage during transsphenoidal surgery. Then we did title and abstract review and full-text examination to determine the selected studies.

### Data abstraction

All of the data were extracted independently by two authors (Tan and Song). The information collected from each study included study and publication year, country, research institution, type, sample size, gender distribution, mean age, outcome, and LD protocol. Any disagreements were resolved by consensus between two investigators.

### Assessment of risk of bias and of quality

Two investigators (Tan and Song) independently assessed the methodological quality of the included RCT according to the Cochrane Collaboration guidelines [17]. Meanwhile, the quality assessment of the 4 cohort studies was conducted according to the Newcastle–Ottawa Scale (NOS), which was shown as a nine-point scale [18]. The scores were 4 for selection quality, 2 for comparability, and 3 for quality of outcome and follow-up adequacy. The studies’ quality was ranked as low (below 3 points), moderate (4–6 points), and high (7–9 points). At last, funnel plots would be used to detect publication bias, if possible. Any disagreements were resolved by consensus between two investigators.

### Statistical analysis

The endpoint of this meta-analysis was data on CSF leakage during the perioperative period of pituitary adenomas transsphenoidal surgery. We performed the analysis using Review Manager Version 5.3.5 software. For dichotomous variables, we calculated the risk ratio (RR) with a 95% confidence interval (CI). We used the Mantel-Haenszel method to calculate the weighted summary RR. Significant RR heterogeneity was tested by calculating the I-squared (I^2^) statistic. Whenever I^2^ was less than 50%, the fixed-effects model results were used; otherwise, the random-effects model results were preferred. What’s more, in order to analyze the sources of heterogeneity, a sensitivity analysis was performed in which one study at a time was removed and the rest analyzed to evaluate whether the heterogeneity could be eliminated by a single study. A *P* value less than 0.05 was considered statistically significant for all of the outcomes.

## Results

### Literature search

**Fig. 1:**
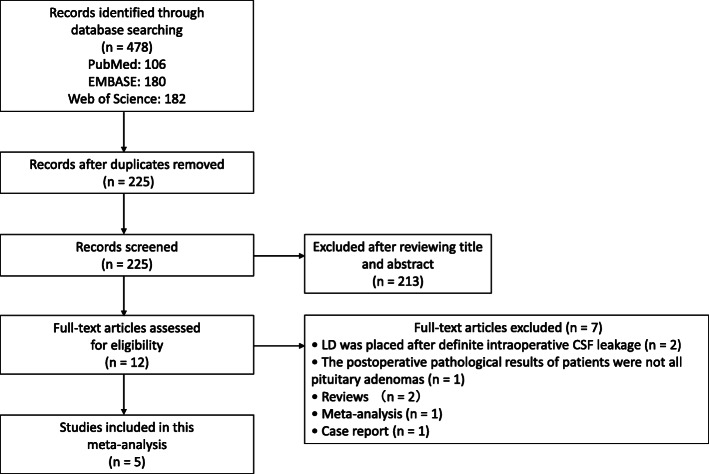
Flowchart of the literature search performed

The entire literature search process is shown in Fig. 1. After a comprehensive literature search in the PubMed, EMBASE, and Web of Science databases, 478 records were identified. After deleting duplicate records, a total of 225 records remained for the title and abstract review. Of these, 12 articles were selected for full-text examination. Two were excluded because LD was placed after a definite intraoperative CSF leakage. One was excluded because the patients’ postoperative pathological results were not all pituitary adenomas. Two were reviews, one was a meta-analysis and one was a case report. Five articles were ultimately included in our study. One was an RCT, and four were cohort studies.

### Characteristics of the included studies

The characteristics of the 5 included articles are shown in Table 1. All of our studies were published from 2006 to 2020, which were conducted by reliable research institutions in five different countries. The studies contained a total of 678 cases. The gender distribution and mean age of the studies were clear except for the study of Sade et al. Three studies had data on both intraoperative and postoperative CSF leakage, 1 only had data on intraoperative CSF leakage, and 1 only had data on postoperative CSF leakage. The studies’ LD protocol were clear.

**Table 1:** Summary of characteristics of the included studies

Study, y	Country	Research institution	Type	Sample size	Gender distribution male/female	Mean age, y	Outcome	LD Protocol
Jonathan 2018 [11]	India	a private, minority-run medical school, hospital and research institute	RCT	60	LD 17/13 no LD 14/16	LD 36.7 ± 12.4 no LD 41.8 ± 11.4	Intraoperative CSF leakage: LD 1/30; no LD 14/30 Postoperative CSF leakage: LD 0/30; no LD 1/30	30 ml CSF at first, then intraoperative drained if necessary, removed immediately after surgery, or kept draining for 5 days if a CSF leakage happened.
Liu 2020 [12]	China	a third-grade class-A hospital	cohort study	189	LD 61/58 no LD 38/32	LD 45.5 ± 17.5 no LD 47.8 ± 15.9	Intraoperative CSF leakage: LD 12/119; no LD 22/70 Postoperative CSF leakage: LD 4/119; no LD 8/70	10–20 ml CSF at each time, removed immediately after surgery, or kept draining 150 ml CSF per day for 3–5 days if a CSF leakage happened.
Mehta 2012 [13]	America	an academic health care center associated with the University of Virginia	cohort study	158	LD 27/17 no LD 46/68	LD 55 ± 15 no LD 47 ± 15	Intraoperative CSF leakage: LD 2/44; no LD 47/114 Postoperative CSF leakage: LD 2/44; no LD 6/114	20 ml CSF at each time for a total of 20–60 ml CSF, removed immediately after surgery.
Sade 2006 [14]	Canada	an acute-care teaching hospital affiliated with McGill University	cohort study	85	Not clear	Not clear	Intraoperative CSF leakage: LD 12/32; no LD 22/53	Infuse 5–25 ml saline, then drained same amount of CSF.
Alharbi 2018 [15]	Saudi Arabia	A tertiary referral hospital	cohort study	186	87/99	50.3 ± 16.1	Postoperative CSF leakage: LD 1/51; no LD 7/135	Drained for 48 h, then removed.

### Data analysis

**Fig. 2:**
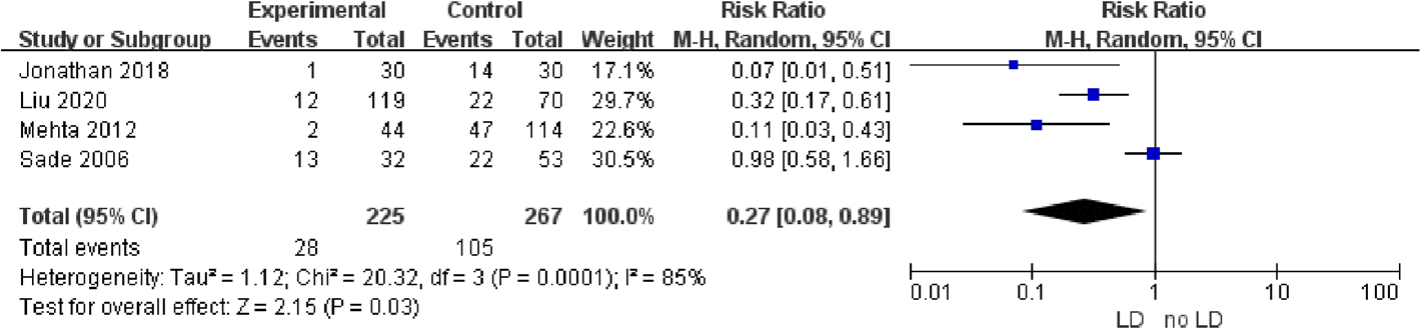
Forest plot analyzing the effect of intraoperative LD in preventing intraoperative CSF leakage

**Fig. 3:**
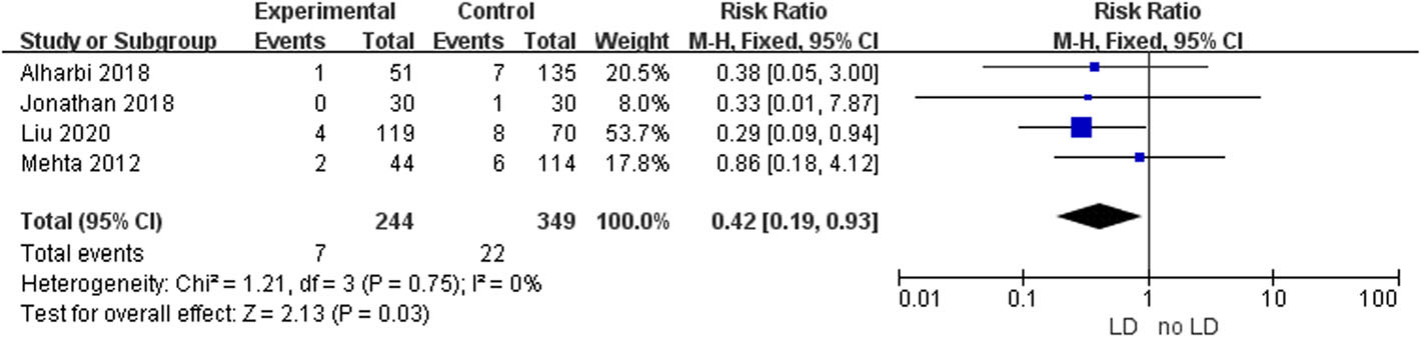
Forest plot analyzing the effect of intraoperative LD in preventing postoperative CSF leakage

**Fig. 4:**
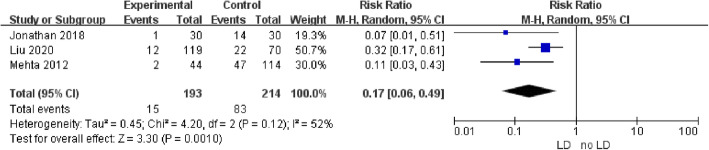
Sensitivity analysis of intraoperative LD in preventing postoperative CSF leakage

Four studies provided data on intraoperative CSF leakage: it occurred in 28 out of 225 (12.4%) cases in the intraoperative LD group, and 105 out of 267 (39.3%) cases in the intraoperative no-LD group. Pooled analysis showed a statistically significant difference in favor of intraoperative LD (RR 0.27; 95% CI 0.08–0.89; *p* = 0.03; Fig. 2). Heterogeneity was statistically significant (I^2^ = 85%, *p* = 0.0001). Four studies provided data on postoperative CSF leakage: it occurred in 7 out of 244 (2.9%) cases in the intraoperative LD group, and 22 out of 349 (6.3%) cases in the intraoperative no-LD group. Pooled analysis showed a statistically significant difference in favor of intraoperative LD (RR 0.42; 95% CI 0.19–0.93; *p* = 0.03; Fig. 3). Heterogeneity was not statistically significant (I^2^ = 0%, *p* = 0.75). A sensitivity analysis was performed to analyze the sources of heterogeneity. When one study was excluded, the heterogeneity (I^2^ = 52%; *p* = 0.12) decreased and was not statistically significant (Fig. 4).

### Risk of bias and quality

**Fig. 5:**
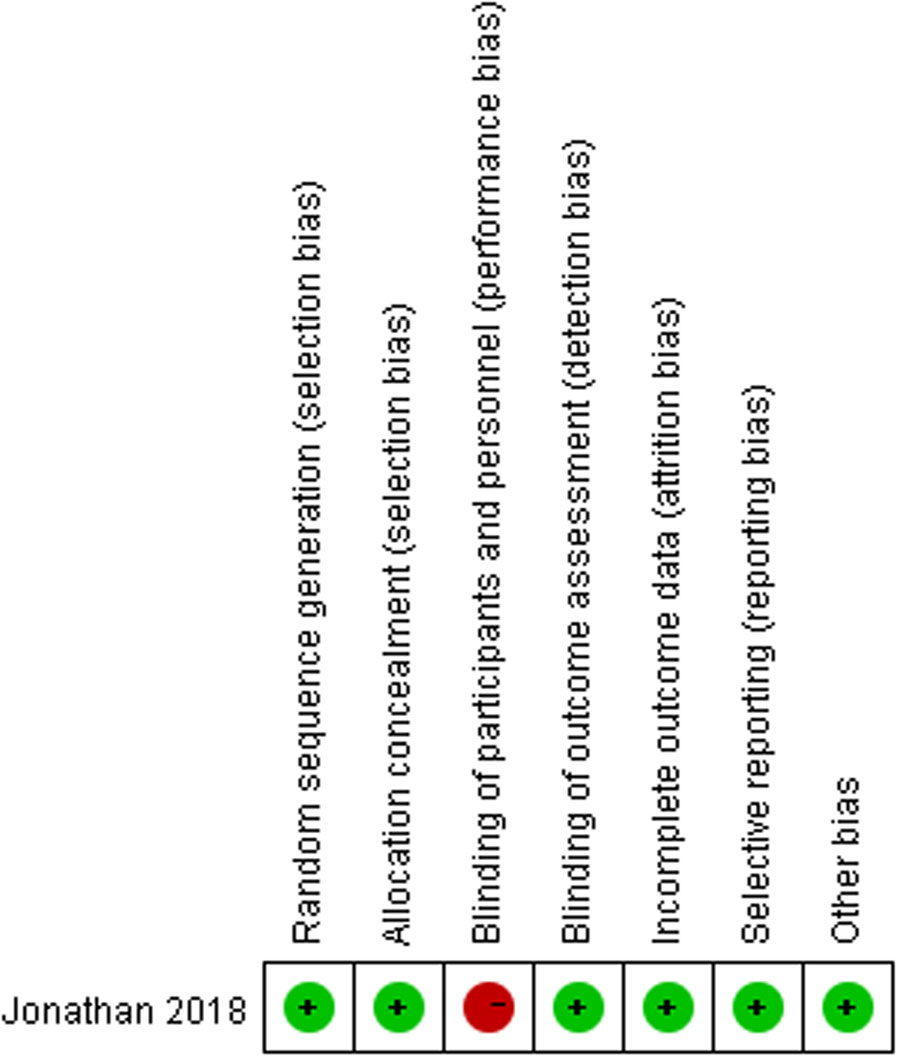
Risk of bias of the included RCT

The Cochrane Collaboration’s tool for assessing the risk of bias is shown in Fig. 5. Performance bias of the 1 RCT was high, the reason was that surgery was not blinded to the operators and patients because it involved preoperative conversations and the signing of surgical consent forms. Thus the 1 RCT was still of high quality. Regarding the literature quality scores, all 4 cohort studies were high quality (rating 7–9 points) as shown in Table 2. Since a small number of articles were included in this study, approaches for detecting publication bias such as funnel plots, would have exhibited limited efficacy. Therefore, publication bias was not assessed.

**Table 2:** Results of quality assessment using the NOS for cohort studies

	Selection				Comparability	Outcome			
Study	Representativeness of the Exposed Cohort	Selection of the Non-Exposed Cohort	Ascertainment of Exposure	Demonstration That Outcome of Interest Was Not Present at Start of Study	Comparability of Cohorts on the Basis of the Design or Analysis	Assessment of Outcome	Was Follow-Up Long Enough for Outcomes to Occur	Adequacy of Follow Up of Cohorts	Quality Score
Liu 2020 [12]	★	★	★	–	★★	★	★	★	8
Mehta 2012 [13]	★	★	★	–	★★	★	★	★	8
Sade 2006 [14]	★	★	★	–	★	★	★	★	7
Alharbi 2018 [15]	★	★	★	–	★	★	★	★	7

## Discussion

To the best of our knowledge, no meta-analysis was previously conducted to explore the relationship between intraoperative LD and CSF leakage rates during the perioperative period of pituitary adenomas transsphenoidal surgery. Thus, the aim of this meta-analysis was to definite whether intraoperative LD can prevent CSF leakage during the perioperative period of pituitary adenomas transsphenoidal surgery. The estimated pooled results showed that intraoperative LD was an effective measure for decreasing CSF leakage rate during pituitary adenomas transsphenoidal surgery. And it worked both intraoperative and postoperative.

CSF leakage during transsphenoidal surgery is usually due to iatrogenic arachnoid injury. Pituitary adenomas can expand the sellar diaphragm, exposing the arachnoid to intraoperative injury, then causing CSF leakage. The force during tumor resection enhanced tension on the arachnoid, which might tear the arachnoid. A tear of arachnoid increased the risk of CSF leakage [19]. Intraoperative LD can reduce strain on the arachnoid, making it less susceptible to puncture [11, 13, 20]. The majority of nonfunctioning macroadenomas with a suprasellar extension may be bounded only by a very thin layer of a normal gland or by arachnoid, and the arachnoid is more likely to tear. Intraoperative LD might be used for their resection [21].

A meta-analysis was published by D’Anza et al. in 2016, their results showed that preoperative or intraoperative LD played no apparent role in preventing postoperative CSF leakage during endoscopic skull base lesions surgery. In their study, the overall postoperative CSF leakage rate was 5.59% (21 of 376 cases), 8.62% (15 of 174 cases) in a preoperative or intraoperative LD group, and 3.97% (6 of 202 cases) in a no-LD group [9]. A cohort study was published by Caggiano et al. in 2018, which included a relatively large sample size of 811 cases. Their results showed that intraoperative LD played no clear role in preventing intraoperative and postoperative CSF leakage during endoscopic endonasal transsphenoidal skull base lesions surgery. In their study, intraoperative CSF leakage occurred in 55.5% of cases (10 of 18) with intraoperative LD and 36.9% of cases (273 of 740) without intraoperative LD. Postoperative CSF leakage occurred in 5% of cases (2 of 38) with intraoperative LD and in 2% of cases (16 of 771) without intraoperative LD [22]. The results of previous studies differed significantly from those of our meta-analysis. The main reason for the differences might be: (1) The pathologic types of skull base lesions were not limited to pituitary adenomas. Resection of lesions such as craniopharyngiomas or suprasellar meningiomas is more difficult than resection of pituitary adenomas. Operators are more likely to damage the arachnoid during surgery, leading to CSF leakage. (2) The meta-analysis of D’Anza et al. [9] included 5 low-quality, small sample studies. The case number in an intraoperative LD group was much less than in a group without intraoperative LD in the study by Caggiano et al. [22]. Therefore, the underrepresentation of the studies and the low comparability of the cases led to different results in our meta-analysis.

The results demonstrated the effective use of intraoperative LD during the perioperative period of pituitary adenomas transsphenoidal surgery. However, high heterogeneity was found in our meta-analysis of intraoperative CSF leakage (RR 0.27; 95% CI 0.08–0.89; *p* = 0.03; I^2^ = 85%; *p* = 0.0001). We could not conduct subgroup analyses or meta-regression due to the few studies included. Thus, we performed a sensitivity analysis to analyze the sources of heterogeneity. When the study of Sade et al. [14] was excluded from this meta-analysis, the heterogeneity (I^2^ = 52%; *p* = 0.12) decreased significantly and was not statistically significant (Fig. 4). We reviewed the study in detail and analyzed the following reasons that might have led to the deviation in the results: (1) Patients were not only diagnosed with pituitary adenoma according to the design of the study. When we extracted data, the baseline data such as gender distribution and mean age were not clear. Besides, all pituitary adenomas had suprasellar extension. These two aspects showed that experimental subjects’ representation was not strong enough. (2) LD was inserted in patients who were thought to be likely to experience intraoperative CSF leakage, the patients underwent selective LD according to the operators’ experience, which meant that the LD group’s representation was not strong enough. (3) Saline infusion was used in some of the patients in the intraoperative LD group, to facilitate the descent of the diaphragm or tumor. Although the infused amount was removed before surgery, venous engorgement continued, and facilitating the descent of the suprasellar portion had a seemingly higher risk of intraoperative CSF leakage and inadvertent arachnoid rupture [23]. We believe that the sensitivity analysis obtained a relativity stable outcome.

In this meta-analysis, a forward association between intraoperative LD and CSF leakage during the perioperative period of pituitary adenomas transsphenoidal surgery was observed, but some limitations still exist: (1) Due to the relativity small number of published studies and sample size, this meta-analysis combining 1 RCT and 4 cohort studies was unable to complete a subgroup analysis, and publication bias was not assessed, which affected the authenticity of the results to a certain extent. (2) Because the interventions in this meta-analysis were different subtypes of surgery, doctors and patients had relativity strong subjectivity in the choice of surgical methods. In addition, the LD devices added an extra cost, increasing the difficulty of random assignment. (3) Although intraoperative LD could reduce strain on the arachnoid, making it less susceptible to puncture. On the contrary, it might conceal the small arachnoid tear that occurred during tumor resection, affecting the judgment of CSF leakage, thus affecting the results of the studies. (4) There were extreme variabilities of the surgical series found in literature, such as tumors’ size, first or second surgery, tumors’ consistency. In this way, the reliability of the results was reduced. (5) LD can reduce CSF leakage, but LD complication rates were reported as 3% for major and 5% for minor complications [24]. Complications included headache, nausea, and vomiting [25, 26]; meningitis and other infections [27, 28]; abducens palsy [29]; intracranial hypotension [30]; cerebellar tonsillar herniation [31]; intracranial venous thrombosis [32]; and lumbar nerve root irritation, retained catheters and pneumocephalus [20]. These complications can lead to higher costs, longer hospital stays, and most importantly, more suffering. (6) Although sensitive analyses were conducted to assess the origin of heterogeneity, heterogeneity across the studies was undeniable.

As far as we know, the suprasellar extension and invasiveness of pituitary adenoma might play a role in CSF leakage development [33]. Unfortunately, only three of our included studies discussed the relationship between the suprasellar extension of pituitary adenoma and perioperative CSF leakage [11–13]. Two of them used Wilson grades to determine the extent of suprasellar extension [12, 13], while one of them just described the tumor with suprasellar extension or with not [11]. Due to the small number of studies and sample size, and the inconsistency of key data, it was of little significance to conduct a meta-analysis to show whether the suprasellar extension of pituitary adenoma played a role in CSF leakage development or not, so we did not conduct it.

Modern skull base reconstruction techniques combined with LD can prevent CSF leakage during the perioperative period of transsphenoidal surgery to a significant extent [34, 35]. Using LD and improving the detection accuracy of intraoperative CSF leakage can reduce skull base reconstruction pressure, especially for young doctors. In general, all of these approaches are designed to improve patient prognosis.

## Conclusion

Although the results of this meta-analysis suggest intraoperative LD can reduce the risk of CSF leakage during the perioperative period of pituitary adenomas transsphenoidal surgery, the available evidence is indefinite. To some extent the results suggest intraoperative LD’s potential positive role. Some possible anatomic mechanisms may explain the results. Further studies that include well-designed prospective, randomized controlled clinical trials are necessary for further verification.

## Data Availability

All data generated or analyzed during this study are included in this published article and its supplementary information files.

## Abbreviations

CSF: Cerebrospinal fluid
LD: Lumbar drainage
PRISMA: Preferred Reporting Items for Systematic Reviews and Meta-Analysis
MeSh: Medical Subject Headings
RCT: Randomized controlled trial
NOS: Newcastle–Ottawa Scale
CI: Confidence interval
RR: Risk ratio
I^2^: I-squared
